# Dietary patterns related to zinc and polyunsaturated fatty acids intake are associated with serum linoleic/dihomo-γ-linolenic ratio in NHANES males and females

**DOI:** 10.1038/s41598-021-91611-7

**Published:** 2021-06-09

**Authors:** Jacqueline Pontes Monteiro, Carlos A. Fuzo, Fábio V. Ued, Jim Kaput

**Affiliations:** 1 Department of Pediatrics and Department of Health Sciences, Faculty of Medicine, Nutrition and Metabolism, University of São Paulo, Avenida Bandeirantes, Bairro Monte Alegre, Ribeirão Preto, SP 3900 Brazil; 2 Department of Clinical Analyses, Toxicology and Food Sciences, School of Pharmaceutics Sciences, University of São Paulo, Ribeirão Preto, SP Brazil; 3 Vydiant, Folsom, CA USA

**Keywords:** Predictive markers, Biomarkers, Medical research

## Abstract

Identifying dietary patterns that contribute to zinc (Zn) and fatty acids intake and their biomarkers that may have an impact on health of males and females. The present study was designed to (a) extract dietary patterns with foods that explain the variation of Zn and PUFAs intake in adult men and women; and (b) evaluate the association between the extracted dietary patterns with circulating levels of serum dihomo-γ-linolenic fatty acid (DGLA) or serum linoleic/dihomo-γ-linolenic (LA/DGLA) ratio in males and females. We used reduced rank regression (RRR) to extract the dietary patterns separated by sex in the NHANES 2011–2012 data. A dietary pattern with foods rich in Zn (1st quintile = 8.67 mg/day; 5th quintile = 11.11 mg/day) and poor in PUFAs (5th quintile = 15.28 g/day; 1st quintile = 18.03 g/day) was found in females (S-FDP2) and the same pattern, with foods poor in PUFAs (5th quintile = 17.6 g/day; 1st quintile = 20.7 g/day) and rich in Zn (1st quintile = 10.4 mg/day; 5th quintile = 12.9 mg/day) (S-MDP2), was found in males. The dietary patterns with foods rich in Zn and poor in PUFAs were negatively associated with serum LA/DGLA ratio. This is the first study to associate the LA/DGLA ratio with Zn and PUFAs related dietary patterns in males and females.

## Introduction

Studies have shown associations between carbohydrate and lipid intakes with the risk for developing non-communicable diseases (NCD)^1–3^, but the micronutrients role in reducing the risk of NCD is understudied. Zinc (Zn), an intracellular metal involved in numerous metabolic processes^4^ has been cited (with iron, vitamin A, folate, and vitamin B12) as one of the five micronutrients of public health importance^5^. Its deficiency affects around 17% of the world’s population^6^. Since multiple physiological functions are affected by Zn deficiency^7^, monitoring individual and population Zn status is crucial for maintenance of health and, in this context, defining new biomarkers of Zn intake is important. People do not eat isolated Zn, but rather complex combinations of nutrient and food components that are interactive or synergistic^8,9^.

The synergetic relationship between Zn and dietary lipids on the risk for NCD has received little attention. Zn and essential fatty acids are involved in transcriptional regulation and their deficiencies present similar clinical features and symptoms, which suggest overlap not only in gene regulation but also in some metabolic pathways related to NCD^10^. Enzymes involved in metabolism of ω-6 series derived from *cis*-linoleic acid (LA, 18:2) and the ω-3 series derived from α-linolenic acid (ALA, 18:3) are metabolized by Zn-containing enzymes^11^. *Cis*-linoleic acid (LA, 18:2) is converted to γ-linolenic acid (GLA, 18:3, n-6) by Δ^6^ desaturase (d-6-d), and GLA is elongated to form dihomo-GLA (DGLA, 20:3, n-6). In this scenario, Zn plays a critical role in regulating the LA/DGLA ratio, which may be a useful indicator for assessing Zn and PUFAs intake^12^.

Dietary patterns offer a perspective different from the traditional single nutrient intake analysis and may be used to formulate more comprehensive dietary recommendations for health and disease prevention or treatment^13–15^. Identifying dietary patterns that contribute to Zn and fatty acids intake and that are related to LA/DGLA ratio may provide a better understanding for the variation in Zn- and PUFA-related metabolites and their influences on health. These metabolites are generated in Zn-dependent reactions and depend upon PUFA concentrations^10–12^, hence looking for associations between these metabolites and dietary patterns that consider Zn or PUFA separately, would lead to false conclusions.

We hypothesized that individuals who have a dietary pattern with foods low in Zn and high in PUFAs would have low values for serum DGLA and high values for LA/DGLA ratio. Conversely, individuals who have a dietary pattern with foods rich in Zn and poor in PUFAs would have high values for serum DGLA and low values for LA/DGLA ratio. Since sex hormones may influence the enzymatic synthesis of long-chain polyunsaturated fatty acids (LC-PUFAs)^17^, we analyzed these associations by sex.

Reduced rank regression (RRR)^16^, a-posteriori approach, was used to (i) extract dietary patterns that could explain as much variation of Zn and PUFAs intake as possible, and (ii) to associate the resultant dietary patterns with serum dihomo-γ-linolenic fatty acid (DGLA) and with serum linoleic/dihomo-γ-linolenic (LA/DGLA) ratio in adult females and males using the NHANES 2011–2012 data.

## Material and methods

### Study population

This retrospective observational cross-sectional study used the National Health and Nutrition Examination Survey (NHANES) data separated by sex. NHANES is conducted every year on individuals of all ages. The NHANES study is conducted according to the Declaration of Helsinki and all procedures involving human subjects are approved by NCHS Research Ethics Review Board (ERB) (Protocol #2011-17)^18^. Details of the design and content of NHANES and public use data files are available on the NHANES website^19^.

Of the 13,431 persons recruited in the NHANES 2011–2012 study, 9756 completed the dietary interview. The analyses in the present study considered females and males, separately. The present study excluded (a) individuals with age lower than 20 years; (b) pregnant women; (c) individuals with missing data for weight, height, PUFAs intake, Zn intake, serum LA, serum DGLA; and (d) under/over-reporters for energy intake.

Race and level of education were considered as confounding variables that may affect serum fatty acid. Hispanic (Mexican American; Other Hispanic) and Non-Hispanic (White, Black, Asian, Other multiracial persons) were included. Information on level of education category was collected as follow: less than 9th grade education, 9–11th grade education (includes 12th grade and no diploma), high school graduate/GED, some college or associates (AA) degree, and college graduate or higher. The survey also determined a family income to poverty ratio (PIR) as a study variable. PIR was calculated by dividing family (or individual) income by the poverty guidelines specific to the survey year; the values ranged from 0 to 5.00. Values greater than 5.00 were coded as “5”. More details on demographic NHANES data are available in^20^ https://wwwn.cdc.gov/nchs/nhanes/2011-2012/DEMO_G.htm.

Confounding variables such as lipid lowering drugs and use of dietary supplement containing the words “micronutrient”, “Zinc”, “fat”, “fatty acid”, “trace”, and “oligoelement,” were extracted from NHANES 2011–2012 population through the Dietary Supplement and Prescription Medication Section (DSQ) questionnaire, which provides personal interview data on use of prescription medications during a one-month period prior to the survey date.

Physical activity was also considered to be a confounding variable. The NHANES variables “Vigorous work activity” and “Vigorous recreational activities” were used in our analysis because this level of activity may have an impact on serum fatty acids. Both variables were coded as “yes = 1” and “No = 2”. The ones who refused to answer were coded as “7” and the ones who did not know their physical activity status as “9”^21^. Additional details are described in https://wwwn.cdc.gov/Nchs/Nhanes/2011-2012/PAQ_G.htm.

### Anthropometric data

Standing height and weight were measured in all NHANES sampled personnel in the mobile examination center according to pre-established methods^22,23^. Body mass index (BMI) was calculated as follows: weight (kilograms)/height (meters squared). BMI may have an impact on serum fatty acids.

### Assessment of dietary intakes

Two dietary interviews were done in all participants in NHANES 2011–2012. The primary dietary interview was administered in person and a follow-up dietary interview was conducted by telephone, 3–10 days after the primary one. The USDA Automated Multiple Pass Method (AMPM) program was used for collecting 24-h dietary recalls to extract a list of all the foods and beverages for each person (i) consumed within a 24-h period; the time of consumption, (ii) the name of the eating occasion, (iii) detailed food descriptions and amounts of the reported foods, (iv) where it was obtained, and (v) whether it was eaten at home. The information was coded and linked to a database of foods and their nutrient composition. Calculations of total daily nutrient intakes were derived from these data. The resulting information provided a description of the food item consumed and the intakes of PUFAs, Zn and energy of the study population. For more detailed information see references^24–27^.

### Food groups

Food items (assessed in grams per day) were assigned to one of the 34 food groups predefined from the USDA’s Food and Nutrient Database for Dietary Studies 2011–2012 which permitted the coding of dietary intake data according to the nutrient profiles and culinary use^28^. The dairy products group was divided into low fat and whole fat and the grain products group was also divided into good source of fiber (> 2.5 g/per serving) and poor source (below or equal 2,5 g/per serving) because these groups may have an impact on serum fatty acids. The groups were: low fat dairy products (G1), whole fat dairy products (G2), red meat (G3), poultry (G4), organ meat (G5), fish (G6), meat, poultry, fish with nonmeat items (G7), frozen and shelf-stable plate meals, soups, and gravies with meat, poultry, fish base, gelatin and gelatin-based drinks (G8), eggs (G9), legumes (G10), nuts and seeds (G11), flour and dry mixes, yeast breads, rolls, quick bread (G12), cakes, cookies, pies, pastries, bars (G13), crackers and salty snacks from grain products (G14), pancakes, waffles, French toast, other grain products (G15), pastas, cooked cereals, rice (G16), cereals, not cooked or non-specified as to cooked (G17), grain mixtures, frozen plate meals, soups (G18), meat substitutes, mainly cereal protein (G19), cereals, grains good source of fiber (G20), cereals, grains poor source of fiber (G21), fruits (G22), fruit juices (G23), starchy vegetables (G24), dark green vegetables (G25), yellow and red vegetables (G26), other vegetables (G27), fats (G28), oils (G29), salad dressing (G30), sugars and sweets (G31), nonalcoholic beverages (G32), alcoholic beverages (G33), formulated nutrition beverages (G34). The multiple source method (MSM) was used to remove within-person variation and then to estimate the usual food group intake^26^.

### Serum Zn

Detailed specimen collection and processing procedures are discussed in the NHANES Laboratory Procedures Manual^29^. All NHANES analysis was conducted in CDC laboratories or by their collaborators or subcontractors. The trace metal vacutainer tubes were used only on the second or later blood draws. No fasting or special diet was required. Pre-screened polyethylene vials and pre-screened 7 mL vacutainers were used for specimen acquisition. In between 0.8 to 2.0 ml of serum was required for analysis. Inductively coupled plasma dynamic reaction cell mass spectrometry (ICP-DRC-MS) was used to measure serum Zn. Normal values for Zn: > 75mcg/dl^30^.

### Serum fatty acids

NHANES serum LA (18:2n-6) (µmol/L) and DGLA (20:3n-6) (µmol/L) fatty acids were measured at the CDC using gas chromatography–mass spectrometry. The detailed procedures are in^31^ https://wwwn.cdc.gov/nchs/data/nhanes/2011-2012/labmethods/FAS_G_MET.PDF. All analysis was conducted in CDC laboratories or by their collaborators or subcontractors. Briefly, a fasting 0.5 ml sample was obtained and a volume of 100 μl was required per analysis. Frozen samples were stored at − 70 °C.

Sequential treatment with mineral acid and base in the presence of heat was used to achieve esterified fatty acids. Total fatty acids were hexane-extracted with an internal standard solution containing stable isotopically-labeled fatty acids for recovery. The extract was converted into pentafluorobenzyl esters. The reaction mixture was injected onto a capillary gas chromatograph column to separate the fatty acids of interest only, and were detected using electron capture negative-ion mass spectrometry. Quantitation was accomplished by comparing the peak area of the analyte in the unknown with the peak area of a known amount in a calibrator solution^31^.

### Statistical analysis and reduced rank regression (RRR)

Reduced rank regression (RRR) analysis^16^ was performed with two response variables (PUFAs intake and Zn intake, in grams per day) and the 34 food groups (intake of each food groups in grams per day) in 1614 participants separated by sex (NHANES 2011–2012). At least five individuals per food group is recommended: this study used 34 food groups and a sample of 170 people would sufficiently powered for the analyses described herein^16^). Since nutrient intake is usually highly correlated with energy intake^32^, we calculated energy-adjusted nutrient intakes, using the regression residual method^32^.

The PROC PLS procedure with the RRR method was done with the SAS Statistical Software Version 9.3 (SAS Institute, Inc. Cary, NC, USA). We applied the RRR method to female and male populations. A detailed description of the method can be found in Hoffmann et al.^16^. Briefly, in RRR analysis, *X*_1_, …, *X*_*n*_ and *Y*_1_, …, *Y*_*m*_ are two sets of variables (predictors and responses). In this study, the predictors *X*_*i*_ are intakes of food groups in grams per day and the responses *Y*_*j*_ are intakes of Zn and PUFAs in grams per day. RRR starts from a linear function of responses called response score that will then be projected onto the space of predictors to produce a factor score, that is, a linear function of predictors. Therefore, RRR extracts successive linear combinations of the predictors, called factors or components^16^. RRR does not describe naturally occurring dietary patterns of the population under study but explains variation in important risk factors^33^ and hence, this is an exploratory approach and dietary patterns are derived from the available data. This approach ignores prior knowledge of population dietary pattern completely in order to extract dietary patterns that explain the variation of Zn and PUFAs intake.

The hypothesis tested here was that intakes of Zn and PUFAs are presumed to be associated with serum PUFAs levels in males and females^10,11^. Dietary Zn and PUFAs were chosen as response variables and the outcomes of interest were serum LA/DGLA ratio and serum DGLA. The dietary pattern score “x” was calculated as the sum of z-standardized intakes (mean = 0, standard deviation = 1) of 34 food groups items multiplied by an individual weight. Further, to reduce the dimensionality of complex data, the score was simplified by including only food groups with high factor loadings > or = to 0.20 and then summing the standardized food group intake while retaining the direction of the factor loading^16^. Food groups with factor loadings equal or higher than 0.2 were considered positive contributors to the patterns, and food groups with factor loadings up to -0.2 were negative contributors to the patterns.

The simplified pattern score defined by the food groups was used to evaluate the association between the dietary pattern and the response variables (partial correlation adjusted by energy intake) but also between the pattern and NHANES 2011–2012 serum PUFAs (outcomes). The individuals simplified score “x” was distributed into quintiles. For descriptive purposes, the median (min–max) intakes of the food groups were calculated across quintiles of the simplified dietary pattern score, as well as anthropometric and serum PUFAs data and frequencies for sample characteristics. Linear trend analysis was used to compare continuous variables in their original scale throughout quintiles, adjusting for energy for food groups. Chi-square test was used to compare categorical variables throughout quintiles. We evaluated the impact of potential effect modifiers on the association between the dietary pattern and the serum PUFAs by linear regression fitting models. We applied unadjusted and adjusted multiple linear regression analysis models to the serum dihomo-γ-linolenic fatty acid and to linoleic/dihomo-γ-linolenic ratio (dependent variables) for NHANES 2011–2012 population separated by sex, with the simplified scores for each dietary pattern as independent variable including the following variables as confounding variables: (model 1) unadjusted; (model 2) adjusted for age, energy intake, poverty / income ratio (PIR), race, physical activity, BMI, level of education, medication use, supplement use as confounding variables. The selection of the covariates was based on theoretical assumptions of their relationship with the outcome.

#### Characterizing under- and over reporters

The Vinken’s equation^34^ was used for predicting total energy expenditure (pTEE) in MJ/day by the following relation:

$$pTEE=7.377-\left(0.073\times age\right)+\left(0.0806\times weight\right)+\left(0.0135\times height\right)-(1.363\times sex)$$

where age is in years, weight is in kg, height is standing height in cm, and sex is 0 for men and 1 for women. McCrory equation described below was used to calculate cut-off points for detecting under- and over reporters^35^.

$$\pm 1SD= \sqrt{\left({CV}_{wEI}^{2}/d\right)+{CV}_{wTEE}^{2}}$$

$$=\sqrt{\left({CV}_{wEI}^{2}/d\right)+{CV}_{wpTEE}^{2}+{CV}_{tmTEE}^{2}}$$

where *CV*_*wEI*_ and *CV*_*wpTEE*_ are the within-subject coefficient of variation obtained from energy intake and from predicted TEE equal to 32.3% and 20.2%, respectively. The number of days of energy intake (*d*) was equal to 2 and the within subject coefficient of variation in measured total energy expenditure (*CV*_*tmTEE*_) was equal to 8.2%^35,36^.

All statistical analyses were performed using R package (version 3.6.0) and SAS software, version 9.4 (SAS Institute Inc., Cary, North Carolina, United States) and a two-sided p-value < 0.05 was considered statistically significant.

### Ethics approval and consent to participate

NHANES study is conducted according to the Declaration of Helsinki and all procedures involving human subjects are approved by NCHS Research Ethics Review Board (ERB) (Protocol #2011–17) (National Center for Health Statistics. National Health and Nutrition Examination Survey. NCHS Research Ethics Review Board Approval [Internet]. [cited 2020 May 26]. Available from: https://www.cdc.gov/nchs/nhanes/irba98.htm). Details of the design and content of NHANES and public use data files are available on the NHANES website (National Center for Health Statistics. National Health and Nutrition Examination Survey [Internet]. [cited 2020 May 26]. Available from: https://www.cdc.gov/nchs/nhanes/Index.htm).

## Results

**Figure 1:**
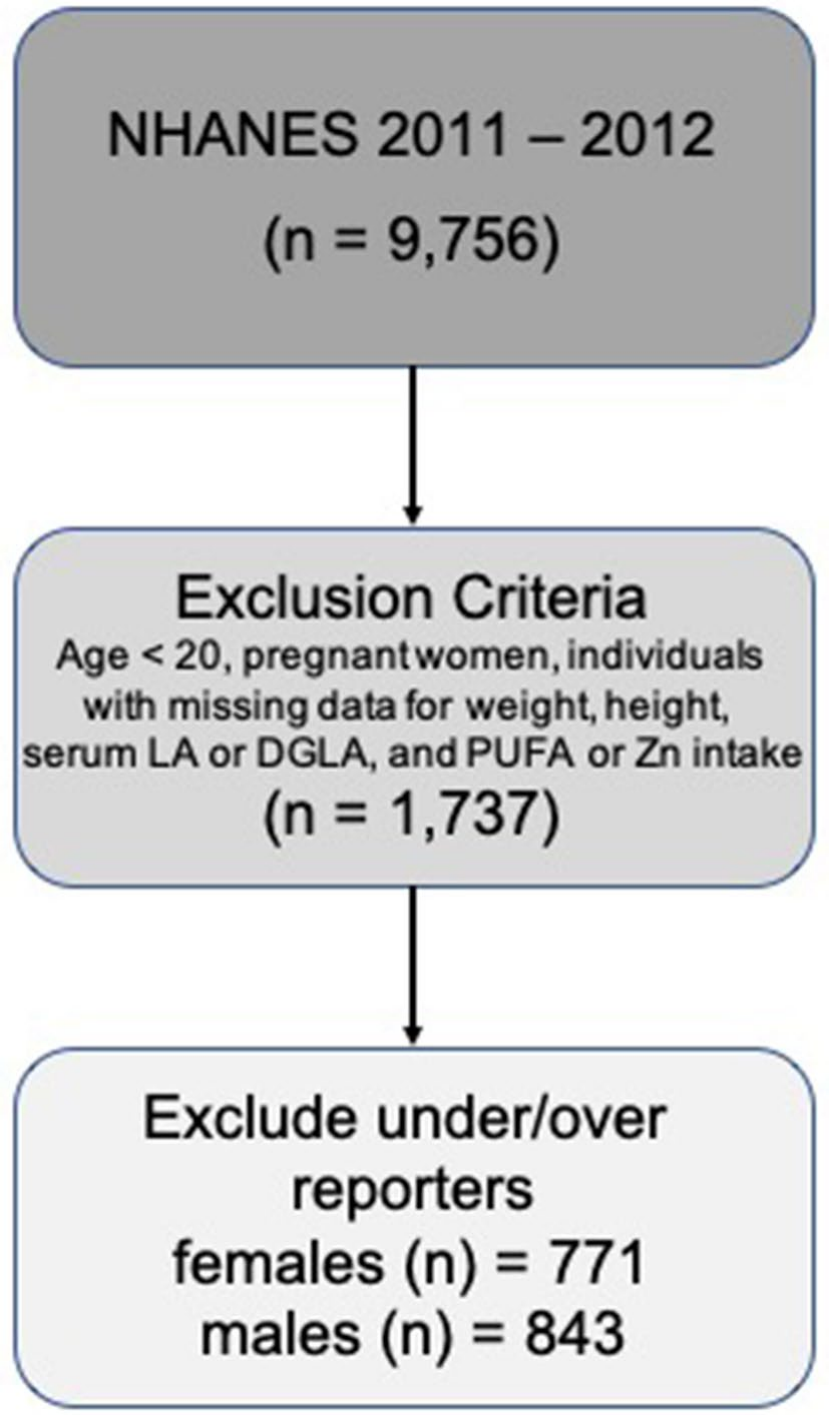
Flow diagram for the selection of the participants in the study from NHANES 2011–2012.

Of the 9756 individuals in the NHANES 2011–2012 database, 1614 participants (771 females and 843 males) met the inclusion criteria (Fig. 1). Demographics characteristics of studied females and males in NHANES 2011–2012 are in Table 1. Females and males were similar regarding age, body mass index, poverty/ income ratio and percentage of individuals using lipid lowering drugs but have small differences in ethnic background and education level. Dietary supplement use was more prevalent in females, and males practice more vigorous activity.

**Table 1:** Demographics characteristics of studied females and males in NHANES 2011–2012.

Demographic variables	NHANES 2011–2012		
	Female (n = 771)	Male (n = 843)	p value
Age (years)	48.6 ± 17.5	47.6 ± 17.7	0.25
Body Mass Index (BMI) (kg/m^2^)	28.3 ± 7.0	27.8 ± 5.6	0.12
Poverty/income ratio (PIR)	2.42 ± 1.61	2.53 ± 1.65	0.20
**Ethnicity**			**0.03**^**1**^
Mexican American (%)	9.7	11.9	–
Other Hispanic (%)	10.9	10.3	–
Non-Hispanic White (%)	37.9	40.9	–
Non-Hispanic Black (%)	24.8	19.2	–
Non-Hispanic Asian (%)	13.7	15.8	–
Other Race—Including Multi-Racial (%)	2.9	1.8	–
**Education level**			** < 0.01**^**1**^
Less than 9th grade (%)	8.2	8.2	–
9–11th grade (%)	11.4	16.5	–
High school graduate (%)	19.2	24.2	–
Some college or AA degree (%)	31.6	25.5	–
College graduate or above (%)	29.6	25.6	–
**Physical activity**			** < 0.01**^**1**^
Vigorous work activity (%)	8.8	24.8	–
Vigorous recreational activities (%)	18.4	25.6	–
**Medication and supplement**			–
Lipid lowering drugs use (%)	17.5	19.3	0.34
Dietary supplement use (%)	27.9	19.7	** < 0.01**^**1**^

^1^p-value refers to differences for the category as a whole.

No statistically significant associations were found between Zn intake alone and DGLA or LA/DGLA ratio without (spearman correlation females r = 0.013, p = 0.71, r = − 0.05, p = 0.14, respectively; and spearman correlation males r = 0.06, p = 0.07, r = − 0.06, p = 0.06, respectively) or with adjustment for energy intake (female r = 0.04, p = 0.19, r = − 0.05, p = 0.12, respectively; male r = − 0.04, p = 0.25, r = 0.03, p = 0.32, respectively).

Since dietary Zn alone was not associated with these circulating fatty acids in men or women, either with or without adjustments for energy intakes, dietary patterns with intake of different ratios of Zn and PUFAs may better explain DGLA or LA/DGLA ratios. Reduced Rank Regression analysis was used to assess whether these two combined response variables (Zinc intake and PUFAs intake) were associated with levels of these specific fatty acids.

### Reduced rank regression

RRR applied to females’ diet intakes extracted a female dietary pattern 1 (FDP1) which had more foods higher in Zn and PUFAs and a female dietary pattern 2 (FDP2) which had more foods higher in Zn and lower in PUFAs (Supplemental Table S1). RRR applied to male population extracted a male dietary pattern 1 (MDP1), which had more foods higher in Zn and PUFAs, and a male dietary pattern 2 (MDP2), which had more foods higher in Zn and lower in PUFAs (Supplemental Table S1).

#### RRR results—female

FDP1 alone explained 39.5% of PUFAs intake variation and 34.3% of Zn intake variation for 771 females meeting the inclusion criteria. FDP1 and FDP2 together explained 49.6% of PUFAs and 46.0% of Zn intake variation. The combined dietary patterns explained 47.8% of all response variables variation.

#### RRR results—male

MDP1 alone explains 37.0% of PUFAs intake variation and 41.0% of Zn intake variation for 843 males meeting the inclusion criteria. MDP1 and MDP2 together explain 47.1% of PUFAs and 50.2% of Zn intakes variation. The combined dietary patterns explained 48.7% of all response variables variation.

### Dietary patterns

**Figure 2:**
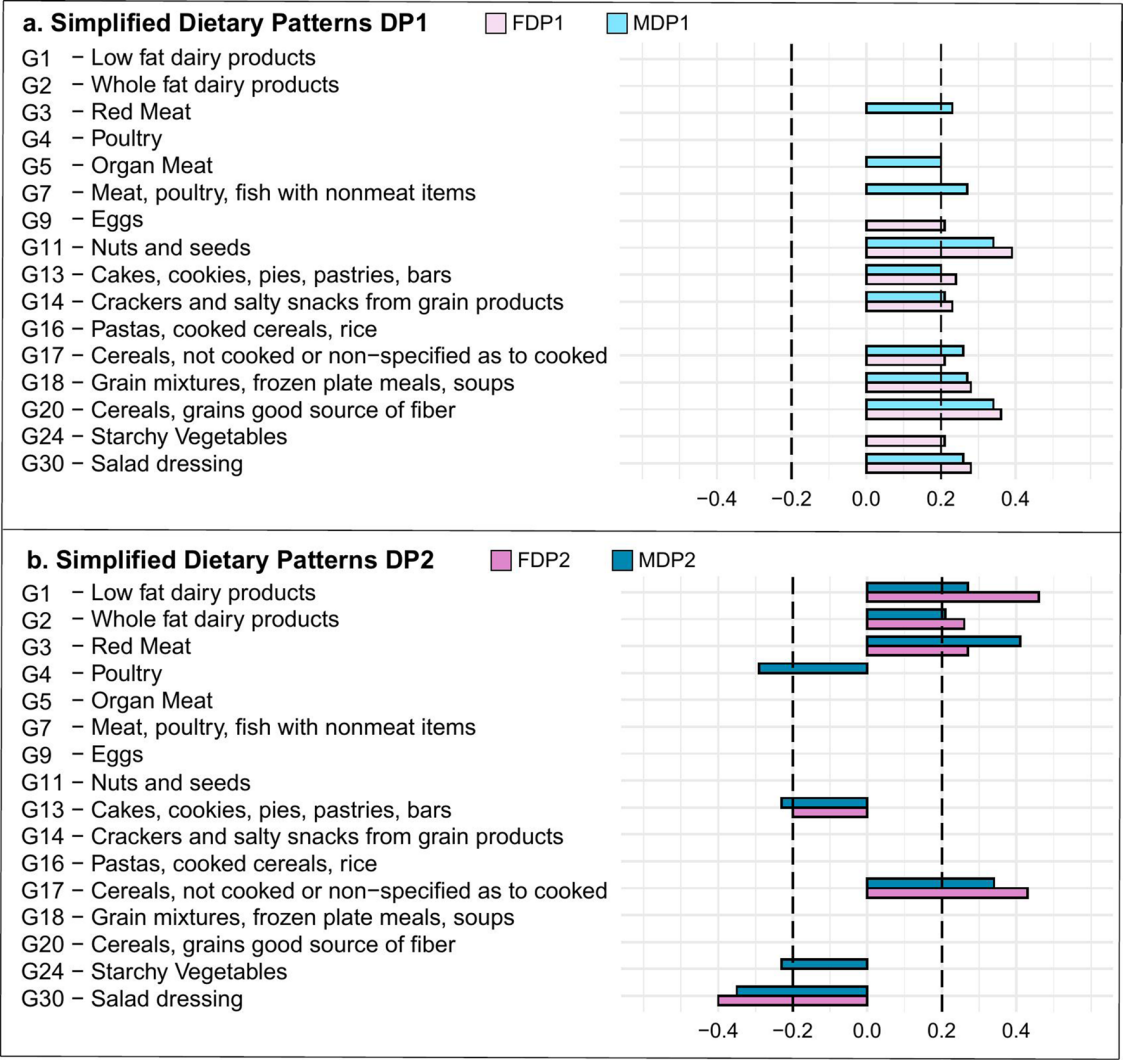
Simplifed Dietary Patterns. RRR loading factors above 0.2 or below − 0.2 shown for each subgroup. (**a**) Simplified dietary patterns 1 for female (FDP1) (light pink) and male (MDP1) (light blue). (**b**) Simplified dietary patterns 2 for female (FDP2) (dark pink) and male (MDP2) (dark blue).

The dietary pattern scores were simplified as described in Methods in female and male populations by using food items with absolute factor loadings equal to or above 0.20 as being significant contributors to a pattern (Fig. 2). These simplified dietary pattern scores were used for all subsequent statistical analysis. Spearman analysis showed a statistically significant correlation between the original dietary scores and the simplified ones in both dietary patterns in female and male, respectively (female: DP1 r = 0.78, p =  < 0.01; DP2 r = 0.85, p =  < 0.01 and male: DP1 r = 0.84, p =  < 0.01; DP2 r = 0.79, p =  < 0.01). The simplified dietary patterns were referred to with an S (females: S-FDP; males: S-MDP). The food groups that contributed most or least to each dietary pattern in NHANES 2011–2012 are in Fig. 2.

PUFAs and Zn intakes (response variables) were modest to moderately correlated with the simplified NHANES 2011–2012 extracted dietary patterns scores after adjustments of energy intake (Table 2).

**Table 2:** Partial correlation between simplified extracted dietary patterns scores and response variables in NHANES 2011–2012.

Number of simplified extracted dietary patterns		Partial correlation (r values)		
		Polyunsaturated fatty acids intake	Zinc intake	p value
**NHANES 2011–2012 (female; n = 771)**				
Simplified Dietary Pattern 1—foods rich in Zn and polyunsaturated fatty acids		0.28	0.10	** < 0.01**
Simplified Dietary Pattern 2—foods poor in polyunsaturated fatty acids and rich in Zn		− 0.33	0.46	** < 0.01**
**NHANES 2011–2012 (male; n = 843)**				
Simplified Dietary Pattern 1—foods rich in Zn and polyunsaturated fatty acids		0.21	0.33	** < 0.01**
Simplified Dietary Pattern 2—foods poor in polyunsaturated fatty acids and rich in Zn		− 0.31	0.42	** < 0.01**

Adjusted for energy intake.

### Specific foods consumed by females

Females with the highest adherence (Table 3, 5th quintile) to the S-FDP1 (foods rich in Zn and in PUFA) were eating more (1) low fat dairy products (that may have contributed to Zn intake), (2) eggs, (3) nuts and seeds, (4) cakes, cookies, pies, pastries, bars, (5) crackers and salty snacks from grain products, (6) cereals not cooked or non-specified as to cooked, (7) grain mixtures, frozen plate meals, soups, (8) cereals and grain products good source of fibers, (9) starchy vegetables, and (10) salad dressing; while females with the highest adherence (5th quintile) to the S-FDP2 (foods rich in Zn and poor in PUFA) were eating more (1) dairy products, (2) red meat, (3) cereals not cooked or non-specified as to cooked, and less (4) cakes, cookies, pies, pastries, bars and (5) salad dressing (Table 3). Serum Zn levels did not change throughout the quintiles (Table 3). The prevalence of females below the lower cutoff for serum Zn concentration was only 3.4% (26 individuals).

**Table 3:** Food groups, response variables intakes and serum Zn across quintiles of the simplified dietary pattern score. Female population NHANES 2011–2012.

	NHANES 2011–2012 Female							
	Simplified dietary pattern 1 (foods rich in Zn and PUFAs)				Simplified dietary pattern 2 (foods rich in Zn and poor in PUFAs)			
	Low adherence 1st quintile	Moderate adherence 3rd quintile	High adherence 5th quintile	p value	Low adherence 1st quintile	Moderate adherence 3rd quintile	High adherence 5th quintile	p value
**Food groups**^**1**^								
Low fat dairy products (g/day)	32.8 (2.7–512)	58.0 (3.1–617)	87.4 (2.4–707)	**0.03**	20.6 (2.4–329)	59.2 (3.1–423)	202 (5.4–707)	** < 0.01**
Whole fat dairy products (g/day)	74.7 (7.1–581)	72.5 (3.7–369)	86.5 (7–588)	0.53	49.1 (4.3–256)	74.0 (4.0–381)	176 (11.8–588)	** < 0.01**
Red meat (g/day)	24.4 (9.2–102)	23.3 (9.1–71)	23.9 (10.4–75)	0.05	20.6 (9.2–55)	24.5 (10–69)	32.6 (11–102)	** < 0.01**
Eggs (g/day)	8.5 (2.5–81)	12.1 (1.2–110)	15.4 (1.5–172)	** < 0.01**	11.2 (1.2–130)	12.4 (2.5–120)	12 (2.6–125)	0.85
Nuts and seeds (g/day)	1.1 (0.2–33)	1.6 (0.2–40)	3.3 (0.2–200)	** < 0.01**	1.2 (0.2–75)	1.4 (0.2–78)	1.8 (0.2–118)	0.12
Cakes, cookies, pies, pastries, bars (g/day)	13.5 (4.4–74)	30.5 (2.3–111)	41.3 (5.3–120)	** < 0.01**	47.7 (5.1–120)	19.9 (5–85)	18.5 (2.3–69)	** < 0.01**
Crackers and salty snacks from grain products (g/day)	4.5 (0.5–25)	7.6 (1.1–51)	18.6 (1–66)	** < 0.01**	7.9 (1.1–66)	7.6 (1.1–55)	6.3 (1–43)	0.06
Cereals, not cooked or non-specified as to cooked (g/day)	1.3 (0.2–35)	1.8 (0.4–59)	2.0 (0.4–113)	** < 0.01**	1.1 (0.4–45)	1.4 (0.3–63)	21.1 (0.4–113)	** < 0.01**
Grain mixtures, frozen plate meals, soups (g/day)	85 (31–271)	120 (29.5–348)	209 (30–517)	** < 0.01**	135.3 (18–517)	133 (34–428)	131 (31–491)	0.45
Cereals, grains, pastas, frozen plate meals, bakery products good source of fiber (g/day)	82.3 (17–284)	141 (28–462)	222 (46–532)	** < 0.01**	159 (17–455)	147 (24–462)	150 (17–532)	0.84
Starchy vegetables (g/day)	22.4 (7–87)	36 (3–145)	42 (8–191)	** < 0.01**	34 (10–137)	32 (7–171)	31 (3–191)	0.20
Salad dressing (g/day)	2.7 (0.5–22)	4.0 (0.2–29)	6.5 (0.5–42)	** < 0.01**	9 (0.4–42)	3.0 (0.2–29)	2.8 (0.5–18)	** < 0.01**
**Response variables**^**1**^								
Zink intake (mg/day)	9.59 (6.2–21)	9.55 (5.15–16)	10.0 (4.3–16.7)	**0.02**	8.67 (4.3–13)	9.41 (6.2–17.3)	11.11 (6.5–20.7)	** < 0.01**
Polyunsaturated fatty acid intake (g/day)	16.15 (8.6–24)	16.8 (10–27.5)	17.41 (9.7–29)	** < 0.01**	18.03 (10.7–31)	16.58 (10–27.5)	15.28 (8.6–29)	** < 0.01**
**Serum zinc**								
Serum Zinc (ug/dl)	84.3 (58–127)	86.6 (69–113)	83.6 (49–123)	0.90	84.1 (49–108)	83.6 (54–119)	83.6 (50–119)	0.86

^1^Differences in quintiles for food and nutrients intakes were tested using linear trend, adjusting for energy. The top number in each cell is the median value and the data in parenthesis is the range.

### Specific foods consumed by males

Males with the highest adherence (Table 4, 5th quintile) to the S-MDP1 (foods rich in Zn and PUFA) were eating more (1) low fat dairy products, (2) red meat, (3) organ meat, (4) meat, poultry, fish with nonmeat items, (5) nuts and seeds, (6) cakes, cookies, pies, pastries, bars, (7) crackers and salty snacks from grain products, (8) cereals not cooked or non-specified as to cooked, (9) grain mixtures, frozen plate meals, soups, (10) cereals and grains products good source of fibers and 11) salad dressing, and less poultry. Males with the highest adherence (5th quintile) to the S-MDP2 (foods rich in Zn and poor in PUFA) were eating less (1) cakes, cookies, pies, pastries, bars, (2) poultry, (3) starchy vegetables, and (4) salad dressing; and more (5) dairy products, (6) red meat, (7) cereals not cooked or non-specified as to cooked, (8) grain mixtures, frozen plate meals, soups, and (9) cereals and grains products good source of fibers (Table 4). Serum Zn did not change throughout the quintiles in males (Table 4). The prevalence of males below the lower cutoff for serum Zn concentration was only 2.5% (21 individuals).

**Table 4:** Food groups, response variables intakes and serum Zn across quintiles of the simplified dietary pattern score. Male population NHANES 2011–2012.

	NHANES 2011–2012 Male							
	Simplified dietary pattern 1 (foods rich in Zn and PUFAs)				Simplified dietary pattern 2 (foods rich in Zn and poor in PUFAs)			
	Low adherence 1st quintile	Moderate adherence 3rd quintile	High adherence 5th quintile	p value	Low adherence 1st quintile	Moderate adherence 3rd quintile	High adherence 5th quintile	p value
**Food groups**^**1**^								
Low fat dairy products (g/day)	25.0 (0.8–580)	30.8 (2.4–652)	107 (2.4–847)	** < 0.01**	22.8 (0.4–392)	29.0 (2.9–469)	219 (2.4–847)	** < 0.01**
Whole fat dairy products (g/day)	62.3 (4.5–536)	67.9 (8.9–533)	84.2 (5.7–650)	0.50	46.3 (4.6–274)	58.9 (4.5–395)	153 (1.3–1072)	** < 0.01**
Red Meat (g/day)	21.8 (6.1–63)	31.6 (6.9–74)	33 (11.5–109)	** < 0.01**	21.6 (6.1–79)	24.9 (6.9–80)	39.4 (10–109)	** < 0.01**
Poultry (g/day)	21.9 (5.1–145)	16.6 (5.2–122)	16.5 (3.0–133)	** < 0.01**	58.8 (5.8–139.6)	16.2 (5.2–133)	13.9 (5.5–125)	** < 0.01**
Organ meat (g/day)	5.7 (1.8–66.3)	9.3 (1.9–83.5)	20.7 (1.8–138)	** < 0.01**	8.0 (1.5–114)	6.7 (2–112)	6.9 (1.1–92.0)	0.29
Meat, poultry, fish with nonmeat items (g/day)	67.8 (27.3–225)	95.4 (26.8–259)	107.6 (31.5–315)	** < 0.01**	96.7 (26.8–223)	74.9 (26.3–248)	86.8 (21.6–256)	0.70
Nuts and seeds (g/day)	1.0 (0.2–29)	1.4 (0.2–49)	9.2 (0.1–118)	** < 0.01**	1.7 (0.04–167)	1.6 (0.1–88)	1.8 (0.1–143)	0.44
Cakes, cookies, pies, pastries, bars (g/day)	13.4 (3–95)	20.5 (4.1–117)	39.7 (4.5–136)	** < 0.01**	43.9 (5.3–141)	20.4 (4.9–111)	16.5 (4.1–88)	** < 0.01**
Crackers and salty snacks from grain products (g/day)	3.5 (0.9–24.5)	5.7 (1.1–56)	13.4 (0.6–80.5)	** < 0.01**	5.8 (0.9–40.4)	5.7 (1.1–55)	5.1 (1.1–56)	0.80
Cereals, not cooked or non-specified as to cooked (g/day)	1.3 (0.3–28)	1.43 (0.3–66)	2.85 (0.2–125)	** < 0.01**	1.18 (0.2–51)	1.33 (0.3–51)	29.3 (0.4–125)	** < 0.01**
Grain mixtures, frozen plate meals, soups (g/day)	83.5 (26.3–367)	158 (28–437)	235 (41–568)	** < 0.01**	123.8 (26–598)	153.5 (29–568)	157 (33–447)	** < 0.01**
Cereals, grains, pastas, frozen plate meals, bakery products good source of fiber (g/day)	78.7 (16.2–289)	142.7 (26.4–488)	250 (22–575)	** < 0.01**	124 (24–469)	157 (17–575)	176.4 (26–586)	** < 0.01**
Starchy vegetables (g/day)	28.0 (5.4–165)	32.4 (6.1–158)	30.0 (4–226)	0.07	57 (7.4–310)	29.2 (5.4–164)	24.6 (6.1–112)	** < 0.01**
Salad dressing (g/day)	2.4 (0.4–34)	4.0 (0.2–37)	7.0 (0.4–69)	** < 0.01**	8.4 (0.5–69)	3.4 (0.4–37)	2.8 (0.4–25)	** < 0.01**
**Response variables**^**1**^								
Zink intake (mg/day)	10.9 (4.4–22)	11.6 (6.0–34)	12.9 (7.8–24)	** < 0.01**	10.4 (5.5–16)	11.4 (4.4–27)	12.9 (8.2–24)	** < 0.01**
Polyunsaturated fatty acid intake (g/day)	18.4 (9.1–27)	18.3 (7.2–30)	20 (9.6–34)	** < 0.01**	20.7 (11–32)	18.8 (9.1–28)	17.6 (7.2–33)	** < 0.01**
**Serum zinc**								
Serum Zinc (ug/dl)	84.9 (60–162)	90.6 (64.2–232)	92.1 (58.6–127)	0.50	91 (62–138)	86.6 (64–121)	83.6 (58.6–119)	0.58

^1^Differences throughout quintiles for food and nutrients intakes were tested using linear trend, adjusting for energy. The top number in each cell is the median value and the data in parenthesis is the range.

One of the objectives of this study was to test associations of dietary patterns with the levels (i.e., outcomes) of serum DGLA and LA/DGLA ratio. In trend analysis, males with the best adherence to a dietary pattern with foods rich in Zn and poor in PUFAs (S-MDP2) had a trend to lowest values for LA/DGLA ratio (p = 0.06). The highest values for serum DGLA and the lowest values for LA/DGLA ratio were observed in females with the best adherence to a dietary pattern with foods rich in Zn and poor in PUFAs (S-FDP2) (Table 5).

**Table 5:** Serum fatty acids across quintiles^1^ of the simplified dietary patterns scores. Female and male populations NHANES 2011–2012.

	Simplified dietary pattern 1				Simplified dietary pattern 2			
	Low adherence 1st quintile	Moderate adherence 3rd quintile	High adherence 5th quintile	p value	Low adherence 1st quintile	Moderate adherence 3rd quintile	High adherence 5th quintile	p value
**Serum fatty acids** **Female (n = 771)**	**Foods rich in Zn and PUFAs**				**Foods rich in Zn and Poor in PUFAs**			
Dihomo-γ-linolenic acid (20:3n-6) (μmol/L)	160 (49.6–391)	154 (42.6–372)	157 (54–465)	0.56	151 (52.6–311)	148.5 (49.6–372)	162.5 (67–465)	** < 0.01**
Linoleic acid/ Dihomo-γ-linolenic acid ratio (μmol/L)	23.6 (9.9–64)	25.9 (10.5–91)	24.3 (8.8–72.5)	0.55	25.7 (11.5–69.3)	26.5 (9.1–72.5)	22.9 (8.9–46.3)	** < 0.01**
**Serum fatty acids** **Male (n = 843*)***	**Foods rich in Zn and PUFAs**				**Foods Rich in Zn and Poor in PUFAs**			
Dihomo-γ-linolenic acid (20:3n-6) (μmol/L)	142 (52–324)	152 (50.7–436)	147 (40.6–426)	0.71	142 (40.6–342)	148 (50.5–436)	149 (59.0–456)	0.14
Linoleic acid/ Dihomo-γ-linolenic acid ratio (μmol/L)	25.1 (9.5–66.2)	24.8 (8.2–57.3)	24.5 (11.9–117)	0.48	25.4 (13.6–117)	24.2 (8.2–66.2)	24.1 (12.5–62)	0.06

^1^Differences throughout quintiles for continuous variables were tested using linear trend. The top number in each cell is the median value and the data in parenthesis is the range.

### Multiple linear regression analysis

#### Dietary patterns in females and biomarkers

Since a relationship was found between dietary patterns and serum DGLA and LA/DGLA through linear trend analysis, we applied unadjusted and adjusted multiple linear regression analysis models. Regression analyses of both the unadjusted (model 1) and adjusted for all confounding variables (model 2) models confirmed that there was a positive association between females with a dietary pattern with foods rich in Zn and poor in PUFA (S-FDP2) and serum DGLA (unadjusted model: R^2^ adjusted 0.016; p < 0.01; adjusted model: R^2^ adjusted 0.168; p < 0.01). There was also a negative association between S-FDP2 and serum LA/DGLA ratio in females (unadjusted model: R^2^ adjusted 0.018; p < 0.01; adjusted model: R^2^ adjusted 0.123; p < 0.01) (Supplemental Table S2).

#### Dietary patterns males and biomarkers

The simplified dietary pattern scores did not show any association with serum DGLA in males with linear regression analyses using either the unadjusted (model 1) or adjusted for all confounding variables (model 2) models. However, a negative association was found between S-MDP2 (foods poor in PUFA and rich in Zn) and serum LA/DGLA ratio (unadjusted model: R^2^ adjusted 0.006; p = 0.01; adjusted model: R^2^ adjusted 0.136; p = 0.01) (Supplemental Table S3).

#### Serum Zn and biomarkers

Serum Zn was not correlated with the LA/DGLA ratio in females and there was a negative trend in males (Spearman: r = − 0.05, p = 0.39; r = − 0.11, p = 0.07, respectively). Serum Zn was statistically correlated with serum DGLA in both females and males (Spearman: female r = 0.13, p = 0.04; male r = 0.14, p = 0.02).

**Figure 3:**
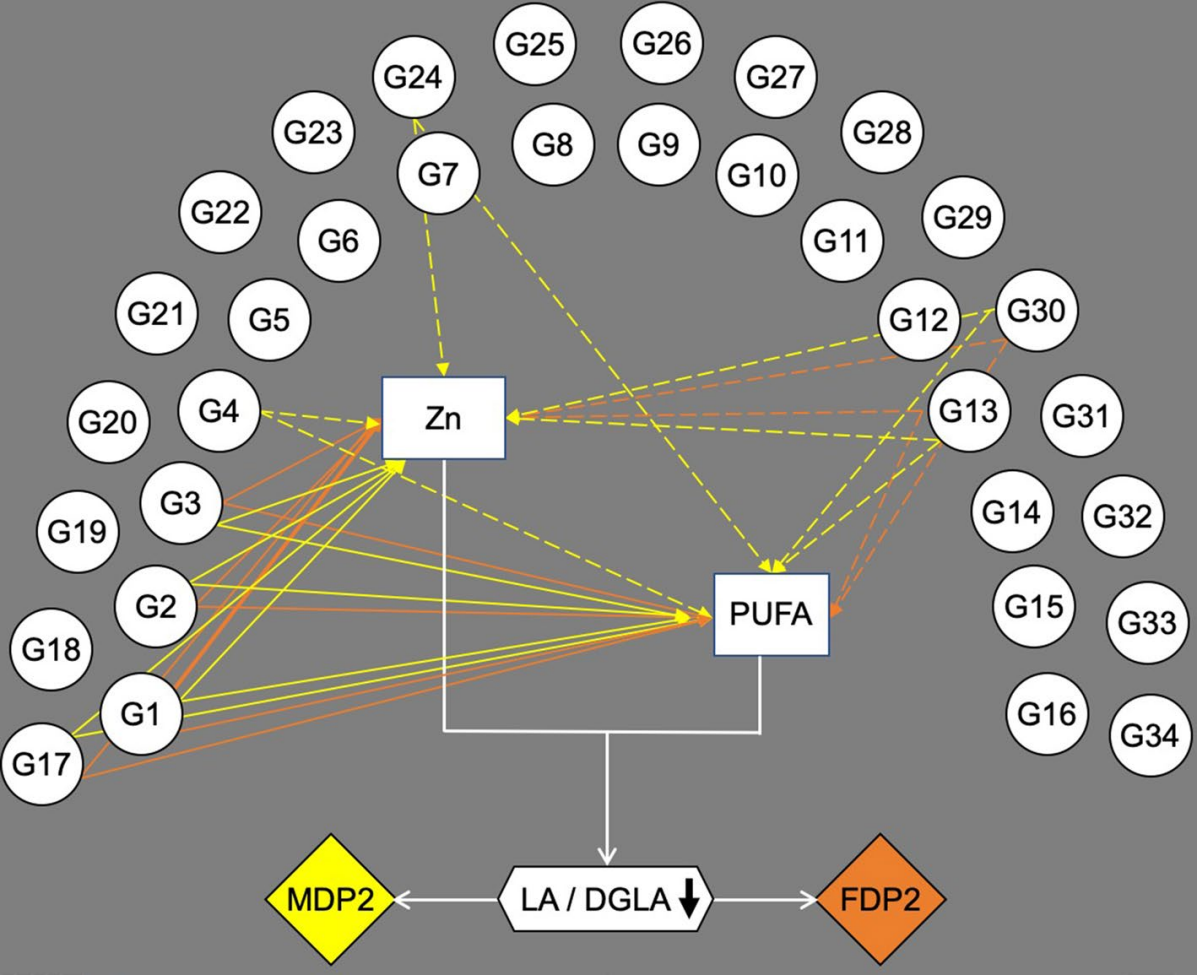
Pipeline and graphical results by which reduced rank regression identified food groups based on intake of zinc (Zn) and polyunsaturated fatty acids (PUFA) followed by multiple linear regression analysis to test for associations of dietary patterns and serum linoleic/dihomo-γ-linolenic ratio (LA/DGLA). Circles with G are the food groups, rectangles are Zn and PUFA intake values, diamonds are dietary patterns 2 for males (MDP2) and females (FDP2). Each dietary pattern (rectangles) is represented by a combination of food groups that explained as much variation of Zn and PUFAs intake. Solid lines are positive and dashed lines are negative associations between food groups and Zn and PUFAs intake. FDP2 and MDP2 (rich in Zn and poor in PUFAs) were associated with low LA/DGLA ratio.

Figure 3 graphically summarizes the analysis pipeline and main results obtained with the reduced rank regression approach.

## Discussion

RRR was used to extract dietary patterns. This revealed dietary patterns that are negatively associated with LA/DGLA ratio. The patterns are similar across males and females, containing foods rich in Zn and poor in PUFAs.

No correlations were found between Zn intake alone and serum DGLA or serum LA/DGLA ratio supporting previous observations that associations between intake of a single nutrient and serum metabolites may potentially be confounded by the effect of dietary patterns^8,9,37^. The amount of Zn and PUFAs intakes found in the present study are in accordance with EAR and RDA^38^ and with other published studies^39,40^.

### Dietary patterns

The two extracted dietary patterns explained 48% and 49% of variation in all response variables in females and males, respectively. Differing percentages of variation in response variables for other dietary patterns have been published^41–44^ but our results were able to measure the extent in variation of the response variables explained by the dietary patterns.

Food groups for RRR analysis were adapted from groups previously defined in the Food and Nutrient Database for dietary studies (FNDDS 2011–2012)^45–47^, which include many foods, mixtures and brand name items. No specific criteria in the literature exists to aggregate food items into food groups although others have used culinary customs, nutrient content^16,48^, or expert opinion^49,50^ to create groups.

The simplified pattern approach was used to construct less data-dependent pattern variables even though it results in a minor loss of information^51^. Absolute factor loadings equal or above 0.20 were considered as significant contributors to a dietary pattern. This value has frequently been used in the context of determining those food items that are significantly associated with factor-analysis-based dietary patterns^49–51^. Less than 10%, of information was lost by using the simplified instead of the original pattern variables. Correlations between response variables and simplified scores were modest to moderate, similar to those found in other studies using RRR analyses of dietary patterns^43,44,50,51^. Our study suggests that the simplified dietary pattern analysis reduced the number of food variables necessary to create a dietary pattern.

The foods in the dietary patterns with more Zn included meat, cereals not cooked, or non-specified as cooked (Zn-fortified foods) which are known to have absorbable Zn^7^. Ready-to-eat breakfast cereals are fortified with Zn, making them a primary Zn source in United States^52^. Lean red meat, whole-grain cereals, provide concentrations of Zn that may vary between 25–50 mg/kg raw weight. The food groups found to contribute to Zn intake in the present study were also found in another study that associated Zn intake and food groups^53^.

Foods rich in PUFAs in the dietary pattern identified in our study were eggs, most vegetable oils, breads, baked goods, margarine, salad dressing and nuts^54^. Individuals who eat salad in USA may also consume more total fat and unsaturated fatty acids and oils in salad dressing^39^.

Our results showed that serum Zn did not correlate with dietary patterns with foods rich in Zn intake which confirms that blood level of Zn is not a good biomarker of its dietary intake^7,53,55–60^.

### Serum zinc status

The present study followed CDC protocols and considered 75 mcg/dL as lower cutoff of fasting Zn concentration^30^. Others have suggested 74 mcg/dL for males and 70 mcg/dL for females for assessing the risk of Zn deficiency^61,62^. The prevalence of individuals with serum Zn deficiency in this study was very low. However, serum Zn (SZCs) has been reported to be higher compared to Zn levels measured in plasma (PZCs)^63^. Nevertheless, PZCs and SZCs are both considered valid estimates of Zn status^64^.

### A possible biomarker of dietary patterns related to Zn intake and PUFAs intakes

The present study is the first one to show that LA/DGLA ratio may be a biomarker of dietary patterns related to Zn and PUFAs intakes in males and females. We did not find statistically significant correlation between Zn intake alone and LA/DGLA ratio or with DGLA alone. Others found inverse associations between Zn intake alone and LA/DGLA ratio and positive associations with DGLA in humans^12,53,56^ and in birds^65,66^. Serum DGLA and the LA/DGLA ratio in the present study were related to short-term Zn and PUFAs intake^67^ in unadjusted and adjusted regression models. Further studies are needed to validate these metabolites as biomarkers and should consider the following factors: plausibility, dose–response, time-response, robustness, reliability, stability and reproducibility. New studies should determine if DGLA or the LA/DGLA ratio are recovery/predictive biomarkers or concentration biomarkers^68^. The present study strengthens the association between LA/DGLA ratio and dietary patterns defined by Zn and PUFAs intakes.

### Serum Zn, DGLA and LA/DGLA ratio

An inverse correlation trend was found between the LA/DGLA ratio and serum Zn (p = 0.07) and a positive correlation between serum Zn and DGLA alone. Others also found a negative correlation trend between LA/DGLA ratio and plasma Zn, but not an association with plasma DGLA alone^53^. On the other hand, another study showed positive correlations between plasma Zn and DGLA and an inverse association between plasma Zn and LA/DGLA ratio^56^.

### Limitations and strengths of the study

The limitations and strengths of the present study are discussed in Box 1. Although a large number of influential studies on US trends in aspects of diet^69^ rely on dietary data collected by the National Health and Nutrition Examination survey, only two 24-h-dietary-recalls are used for data collection. The lack of the usual third recall or the use of other methods decreases accuracy and might affect variation in the estimates of Zn and PUFAs intakes with subsequent alteration in dietary patterns. These effects may be mitigated by the use of the United States Department of Agriculture’s 5-step Automated Multiple-Pass Method which has been shown to reduce bias in dietary intake data^70^.

The present study excluded under/over-reporters that could alter outcomes of the analysis. Several approaches have been suggested to adjust for under/over-reporting^36^. However, excluding large amounts of data that fall above or below the cutoff and a large residual unexplained variation in basal metabolic rate (BMR) determination may decrease the accuracy of the results^71^. Despite the above disadvantages, studies still indicate that implausible reporters be excluded from the analysis^72^.

Correlating diet intake with biomarkers is considered a solution to the limitations of dietary recall methods^68^. However, dietary intake biomarkers also have limitations^73,74^. Indices of nutrient exposure may not necessarily reflect nutrient status, which, in turn, may not necessarily reflect the impact or function of that nutrient^75^. Hence, the analysis pipeline was used to determine whether an association between the dietary patterns and the LA/DGLA ratio exists. In addition, using the LA/DGLA ratio in nutritional epidemiology studies as a biomarker should be preceded by an assessment of its validity, replicability, ability to detect changes, and suitability for the population under study.

RRR shares a number of limitations with other data-driven approaches, one of which is that any identified food intake pattern may be specific to the population under study^17^. This can partially be addressed by validation in a different population^8^ and across different ethnic groups, separated by sex or regardless of sex. Finally, although the models were adjusted by relevant confounders, the selection of the covariates was based on theoretical assumptions, and we cannot rule out other uncontrolled potential confounding factors.

The strengths of the study were that (i) dietary patterns were analyzed instead of individual nutrients which is generally more directly related to dietary recommendations. (ii) Zn and PUFAs intakes were adjusted based on energy intakes which reduces the 24-HDR measurement errors^76–79^; (iii) the study had a reasonable sample size despite the exclusion of under/over-reports—a sample size of at least five individuals per food group is recommended (our study used 34 food groups and a sample of 170 people would have been sufficient^80^); (iv) associations on simplified dietary patterns reduce dimensionality of complex data.

Box 1: Strengths and limitations

**Table:** 

Limitations
The followings are the limitations of the study:
(1) NHANES 2011–2012 used only two 24-h-dietary-recalls (24-HDR). This might have affected the percentage of variation of Zn and PUFAs intake that the dietary patterns explained
(2) The present study excluded under/over-reporters what could have misled the study results
(3) Several interindividual factors can operate and generate variation in LA/DGLA ratio levels, which does not reflect solely differences in dietary intake
(4) RRR shares a number of limitations with the data-driven approaches, including that the identified food intake patterns are specific to the population under study
(5) The regression models were adjusted by relevant confounders and the selection of the covariates was based on theoretical assumptions, and we cannot rule out other uncontrolled potential confounding factors
**Strengths**
The followings are the strengths of the study:
(1) United States Department of Agriculture’s 5-step Automated Multiple-Pass Method has been shown to reduce bias in dietary intake data
(2) Reasonable sample size even after under/over-reporters exclusion. Excluding implausible reports resulted in a dataset of much higher quality according to literature
(3) Energy adjustments were made for Zn and PUFAs intakes which substantially weakened the impact of the 24-HDR measurement error on total nutrient intakes
(4) We reduced the dimensionality of data by constructing simplified dietary patterns
(5) Epidemiologic analyses based on foods, as opposed to nutrients, are generally most directly related to dietary recommendations

## Conclusions and future considerations

This is the first study to associate LA/DGLA ratio with dietary patterns related to Zn and PUFAs intake. Two dietary patterns were negatively associated with serum LA/DGLA ratio. The patterns are similar across males and females, containing foods rich in Zn and poor in PUFAs. Further intervention or observational studies should be done to validate results. These future studies should also consider the integration of omics data to provide insights into the metabolism mechanisms and interference of genetic profile^81–83^.

## Supplementary Information

Supplementary Information.

## Data Availability

Data described in the manuscript, code book, and analytic code are available from the corresponding author on request.

## Abbreviations

24-HDR: 24-Hour-dietary-recalls
AA: Associate in Arts
ALA: α-Linolenic acid
AMPM: Automated Multiple Pass Method
BMI: Body mass index
BMR: Basal metabolic rate
CDC: Centers for Disease Control and Prevention
d-6-d: Δ6 Desaturase
DGLA: Dihomo-γ-linolenic fatty acid
DSQ: Dietary Supplement and Prescription Medication Section
DP: Dietary pattern
EAR: Estimated Average Requirement
ERB: Ethics Review Board
FDP1: Female dietary pattern 1
FDP2: Female dietary pattern 2
FNDDS: Food and Nutrient Database
GED: General Educational Development
GLA: γ-Linolenic acid
ICP-DRC-MS: Inductively coupled plasma dynamic reaction cell mass spectrometry
LA: Linoleic acid
LA/DGLA: Linoleic/Dihomo-γ-linolenic ratio
MDP1: Male dietary pattern 1
MDP2: Male dietary pattern 2
MSM: Multiple source method
NCD: Non-communicable diseases
NCHS: National Center for Health Statistics
NHANES: National Health and Nutrition Examination Survey
PFBBr: Pentafluorobenzyl bromide
PIR: Poverty income ratio
pTEE: Predicting total energy expenditure
PUFAs: Polyunsaturated fatty acids
PZCs: Plasma Zn concentrations
RDA: Recommended Dietary Allowances
RRR: Reduced rank regression
S-FDP1: Simplified female dietary pattern 1
S-FDP2: Simplified female dietary pattern 2
S-MDP1: Simplified male dietary pattern 1
S-MDP2: Simplified male dietary pattern 2
SZCs: Serum Zn concentrations
USDA: U.S. Department of Agriculture
Zinc: Zn
