# Supplementary material for: Dietary patterns related to zinc and polyunsaturated fatty acids intake are associated with serum linoleic/dihomo-γ-linolenic ratio in NHANES males and females
Source: Sci Rep. 2021 Jun 9;11:12215. doi: 10.1038/s41598-021-91611-7 (PMC8190411; doi:10.1038/s41598-021-91611-7)
Supplement: Supplementary file 1 — Supplementary Information. [file 41598_2021_91611_MOESM1_ESM.docx]

**Dietary patterns related to zinc and polyunsaturated fatty acids intake are associated with serum linoleic/dihomo-γ-linolenic ratio in NHANES males and females**

**Jacqueline Pontes Monteiro ^1*^, Carlos A Fuzo ^2^, Fábio V Ued ^1^, Jim Kaput ^3^**

^1^ Department of Pediatrics and Department of Health Sciences, Faculty of Medicine, Nutrition and Metabolism, University of São Paulo, Ribeirão Preto, SP, Brazil (JPM, FVU)

^2^ Department of Clinical Analyses, Toxicology and Food Sciences, School of Pharmaceutics Sciences, University of São Paulo, Ribeirão Preto, SP, Brazil (CAF)

^3^ Vydiant, Sacramento, CA, USA (JK)

* **Corresponding Author**: Jacqueline Pontes Monteiro. Avenida Bandeirantes, 3900. Bairro Monte Alegre. Ribeirão Preto, SP, Brazil. Postal code: 14.040-900. Phone: +55(16) 991548893. e-mail: jacque160165@gmail.com

Supplemental Table 1: Food groups with the loading values for each dietary pattern (factors) in female and male population NHANES 2011-2012.

|  | **NHANES 2011-2012**  **(female)** | | **NHANES 2011-2012**  **(male)** | |
| --- | --- | --- | --- | --- |
| **Food Groups** | **Dietary pattern 1** | **Dietary pattern 2** | **Dietary pattern 1** | **Dietary pattern 2** |
|  |  |  |  |  |
| Low fat dairy products  (G1) | 0.13 | **0.46^1^** | 0.17 | **0.27^1^** |
| Whole fat dairy products  (G2) | 0.14 | **0.26^1^** | 0.19 | **0.21^1^** |
| Red Meat  (G3) | 0.19 | **0.27^1^** | **0.23^1^** | **0.41^1^** |
| Poultry  (G4) | 0.11 | -0.16 | -0.01 | **-0.29^1^** |
| Organ Meat  (G5) | 0.19 | 0.02 | **0.20^1^** | -0.01 |
| Fish  (G6) | 0.12 | -0.14 | 0.01 | -0.12 |
| Meat, poultry, fish with nonmeat items  (G7) | 0.15 | 0.01 | **0.27^1^** | 0.06 |
| Frozen and shelf-stable plate meals, soups, and gravies with meat, poultry, fish base, gelatin and gelatin-based drinks  (G8) | 0.11 | 0.14 | 0.06 | 0.11 |
| Eggs  (G9) | **0.21^1^** | -0.04 | 0.18 | -0.13 |
| Legumes  (G10) | 0.09 | 0.08 | 0.08 | 0.18 |
| Nuts and seeds  (G11) | **0.39^1^** | -0.06 | **0.34^1^** | -0.13 |
| Flour and dry mixes, yeast breads, rolls, quick bread  (G12) | 0.16 | 0.01 | 0.18 | -0.01 |
| Cakes, cookies, pies, pastries, bars  (G13) | **0.24^1^** | **-0.20^1^** | **0.20^1^** | **-0.23^1^** |
| Crackers and salty snacks from grain products  (G 14) | **0.23**^1^ | -0.19 | **0.21^1^** | -0.18 |
| Pancakes, waffles, French toast, other grain products  (G 15) | 0.07 | -0.07 | 0.11 | -0.07 |
| Pastas, cooked cereals, rice  (G16) | -0.01 | 0.16 | -0.14 | 0.18 |
| Cereals, not cooked or non-specified as to cooked  (G17) | **0.21^1^** | **0.43^1^** | **0.26^1^** | **0.34^1^** |
| Grain mixtures, frozen plate meals, soups  (G18) | **0.28^1^** | 0.03 | **0.27^1^** | 0.09 |
| Meat substitutes, mainly cereal protein  (G19) | 0 | 0 | 0 | 0 |
| Cereals, grains good source of fiber  (G 20) | **0.36^1^** | 0.15 | **0.34^1^** | 0.13 |
| Cereals, grains poor source of fiber  (G21) | 0.18 | 0.02 | 0.09 | 0.01 |
| Fruits  (G 22) | 0.03 | 0.12 | 0.05 | 0.08 |
| Fruit Juices  (G 23) | 0.06 | -0.06 | 0.04 | -0.13 |
| Starchy Vegetables  (G 24) | **0.21^1^** | -0.10 | 0.18 | **-0.23^1^** |
| Dark green vegetables  (G 25) | 0.07 | 0.06 | 0.04 | 0.05 |
| Yellow and red vegetables  (G 26) | 0.10 | -0.00 | 0.06 | -0.09 |
| Other vegetables  (G 27) | 0.09 | -0.02 | 0.12 | 0.07 |
| Fats  (G 28) | 0.11 | -0.11 | 0.14 | -0.05 |
| Oils  (G 29) | 0.02 | 0.02 | -0.00 | 0.07 |
| Salad dressing  (G 30) | **0.28^1^** | **-0.40^1^** | **0.26^1^** | **-0.35^1^** |
| Sugars and sweets  (G 31) | 0.09 | -0.02 | 0.16 | -0.04 |
| Nonalcoholic beverages  (G 32) | 0.11 | -0.16 | 0.09 | -0.16 |
| Alcoholic beverages  (G 33) | 0.06 | -0.01 | 0.08 | -0.04 |
| Formulated nutrition beverages  (G 34) | 0.04 | 0.07 | 0.09 | -0.02 |

**^1^** Food groups contributors to the patterns, with factor loadings ≥ 0.2 or ≥ -0.2.

Supplemental Table 2: Linear regression^1^ for serum fatty acids and simplified dietary patterns scores in female population NHANES 2011-2012.

|  | **Unadjusted Model** | | | | | **Adjusted Model ^2^** | | | | |
| --- | --- | --- | --- | --- | --- | --- | --- | --- | --- | --- |
|  | R^2^ | R^2^ adjusted | Beta | F | **ANOVA**  **p value** | R^2^ | R^2^ adjusted | Beta | F | **ANOVA**  **p value** |
| **Serum Dihomo-γ-linolenic fatty acid** | | | | | | | | | | |
| **Dietary Patterns**  **Female** |  | | | | | | | | | |
| 1 – foods rich in zinc and polyunsaturated fatty acids | 0.000088 | -0.001211 | -0.00154 | 0.07 | 0.79 | 0.178963 | 0.158437 | -0.00445 | 8.72 | 0.82 |
| 2 – foods poor in polyunsaturated fatty acids and rich in zinc | 0.017828 | 0.016551 | 0.026671 | 13.9 | **< 0.01** | 0.188583 | 0.168298 | 0.020860 | 9.29 | **< 0.01** |
| **Serum Linoleic/Dihomo-γ-linolenic ratio** | | | | | | | | | | |
| **Dietary Patterns**  **Female** |  | | | | | | | | | |
| 1 – foods rich in zinc and polyunsaturated fatty acids | 0.000672 | -0.000627 | 0.003840 | 0.52 | 0.47 | 0.137130 | 0.115558 | 0.014095 | 6.35 | 0.42 |
| 2 – foods poor in polyunsaturated fatty acids and rich in zinc | 0.020184 | 0.018910 | -0.025601 | 15.84 | **< 0.01** | 0.144490 | 0.123103 | -0.021731 | 6.75 | **< 0.01** |

^1^ Serum fatty acids as dependent variable and simplified dietary factors as independent variable. Unadjusted and adjusted^2^ for all confounding variables (age, energy intake, poverty: income ratio - PIR, race, physical activity, BMI, level of education, medication use, supplement use).

Supplemental Table 3: Linear regression^1^ for serum fatty acids and simplified dietary patterns scores in male population NHANES 2011-2012.

|  | **Unadjusted Model** | | | | | **Adjusted Model ^2^** | | | | |
| --- | --- | --- | --- | --- | --- | --- | --- | --- | --- | --- |
|  | R^2^ | R^2^ adjusted | Beta | F | **ANOVA**  **p value** | R^2^ | R^2^ adjusted | Beta | F | **ANOVA**  **p value** |
| **Serum Dihomo-γ-linolenic fatty acid** | | | | | | | | | | |
| **Dietary Patterns**  **Male** |  | | | | | | | | | |
| 1 – foods rich in zinc and polyunsaturated fatty acids | 0.001706 | 0.000518 | 0.006168 | 1.43 | 0.23 | 0.168641 | 0.150286 | -0.01166 | 9.19 | 0.27 |
| 2 – foods poor in polyunsaturated fatty acids and rich in zinc | 0.004413 | 0.003229 | 0.011577 | 3.72 | 0.05 | 0.166104 | 0.147694 | 0.00717 | 9.02 | 0.06 |
| **Serum Linoleic/Dihomo-γ-linolenic ratio** | | | | | | | | | | |
| **Dietary Patterns**  **Male** |  | | | | | | | | | |
| 1 – foods rich in zinc and polyunsaturated fatty acids | 0.001058 | -0.000129 | -0.004274 | 0.89 | 0.34 | 0.151736 | 0.133008 | 0.012241 | 8.10 | 0.34 |
| 2 – foods poor in polyunsaturated fatty acids and rich in zinc | 0.007460 | 0.006280 | -0.013245 | 6.32 | **0.01** | 0.154576 | 0.135911 | -0.014619 | 8.28 | **0.01** |

^1^ Serum fatty acids as dependent variable and simplified dietary factors as independent variable. Unadjusted and adjusted^2^ for all confounding variables (age, energy intake, poverty: income ratio - PIR, race, physical activity, BMI, level of education, medication use, supplement use).
